# High Diversity and Novel Enteric Viruses in Fecal Viromes of Healthy Wild and Captive Thai Cynomolgus Macaques (*Macaca fascicularis*)

**DOI:** 10.3390/v11100971

**Published:** 2019-10-22

**Authors:** Vorthon Sawaswong, Elizabeth Fahsbender, Eda Altan, Taratorn Kemthong, Xutao Deng, Suchinda Malaivijitnond, Sunchai Payungporn, Eric Delwart

**Affiliations:** 1 Vitalant Research Institute, San Francisco, CA 94118, USA; vorthon007.giftedcru@gmail.com (V.S.); efahsbender@vitalant.org (E.F.); EAltan@vitalant.org (E.A.); xdeng@vitalant.org (X.D.); 2 Department of Biochemistry, Faculty of Medicine, Chulalongkorn University, Bangkok 10330, Thailand; sp.medbiochemcu@gmail.com; 3 Department of Laboratory Medicine, University of California San Francisco, San Francisco, CA 9413, USA; 4 National Primate Research Center-Chulalongkorn University, Saraburi 18110, Thailand; taratorn.kem@gmail.com (T.K.); suchinda.m@chula.ac.th (S.M.); 5 Center of Excellence in Systems Biology, Chulalongkorn University (CUSB), Bangkok 10330, Thailand

**Keywords:** long-tailed macaque, virome, high-throughput sequencing, viral discovery

## Abstract

Cynomolgus macaques are common across South East Asian countries including Thailand. The National Primate Research Center of Thailand, Chulalongkorn University (NPRCT-CU) captures wild-borne cynomolgus macaque for research use. Limited information is available on the enteric viruses and possible zoonotic infections into or from cynomolgus macaques. We characterized and compare the fecal virome of two populations; healthy wild-originated captive cynomolgus macaques (*n* = 43) reared in NPRCT-CU and healthy wild cynomolgus macaques (*n* = 35). Over 90% of recognized viral sequence reads amplified from feces were from bacterial viruses. Viruses from seven families of mammalian viruses were also detected (*Parvoviridae, Anelloviridae, Picornaviridae, Adenoviridae, Papillomaviridae, Herpesviridae,* and *Caliciviridae*). The genomes of a member of a new picornavirus genus we named *Mafapivirus*, a primate chapparvovirus, and a circular Rep-encoding single-strand (CRESS) DNA virus were also characterized. Higher abundance of CRESS DNA viruses of unknown tropism and invertebrate-tropic ambidensovirus were detected in wild versus captive macaques likely reflecting dietary differences. Short term rearing in captivity did not have a pronounced effect on the diversity of mammalian viruses of wild cynomolgus macaques. This study is the first report of the fecal virome of cynomolgus macaques, non-human primates frequently used in biomedical research and vaccination studies.

## 1. Introduction

The virome is the community of viruses found in a particular ecosystem [1]. Viromes characterized from animals and human are comprised of both prokaryotic and eukaryotic viruses [2]. Commensal bacteriophages, which make up the major fraction of the fecal virome, can modulate the microbial community in the host body and influence host immunity [2,3,4,5]. Although typically a smaller fraction of the enteric virome, mammalian viruses may cause diseases such as diarrhea resulting in malnutrition and dehydration [6]. Deep sequencing of wild animal fecal viromes also unveiled many eukaryotic viruses whose pathogenicity, if any, remain mostly unknown [7,8,9,10]. In the past, emergences of human infectious diseases have been initiated by zoonotic viruses originating from bats, rodents, and non-human primates [6,11,12]. Ebola virus likely from bats [13], human immunodeficiency virus (HIV) from chimpanzees [14], and the Middle East respiratory syndrome coronavirus (MERS-CoV) from camels [15,16,17,18], have caused very large economic and public health disruptions. Therefore, it is important to identify the viruses within animals with the potential to spill over into human and result in pathogenic infections. Such zoonoses may take different routes including fecal-oral transmission [19]. Outbreaks of zoonotic enteric viruses belonging to the families of *Picornaviridae*, *Adenoviridae*, *Caliciviridae*, and *Reoviridae* cause important enteric diseases in humans [20]. Moreover, alteration of enteric virome in humans also affect bacterial microbiome stability and influence diseases such as inflammatory bowel disease and ulcerative colitis [21,22]. Studies of intestinal and fecal bacterial communities have received much attention relative to that of the gut virome [23].

Cynomolgus macaque, a non-human primate species widely distributing across Southeast Asian countries [24] have long been used for biological research [25] including on influenza virus [26], Ebola virus [27], and simian/human immunodeficiency virus (SIV/HIV) [28]. The National Primate Research Center of Thailand–Chulalongkorn University (NPRCT-CU), maintains a colony of cynomolgus macaques captured from disturbed natural habitats. Although well-established biosecurity protocols are used to screen infectious viruses such as herpes B virus, simian retrovirus (SRV), simian immunodeficiency virus (SIV), simian-T-lymphotropic viruses (STLV) and foamy virus that might cause a sporadic outbreaks, the transmission of other viruses from wild-originating macaques remains possible [29]. In addition, captivity may also influence gut microbiome and virome. A recent study illustrated that replacing the gut microbiome of inbred laboratory mice with that of wild mice restored their immune responses to better mimic those of wild animals [30]. Here, we characterized and compared the fecal virome of wild and captive macaques and identified novel macaque viruses.

## 2. Materials and Methods

### 2.1. Study Cohort

The cynomolgus macaque (*Macaca fascicularis*) cohort (*n* = 78) was comprised of two colonies, wild macaques (Wild, *n* = 35) captured from natural habitat located in Wat Tham Praporthisat (PPT), Saraburi (GPS: 14° 34’N, 101° 08’E) and wild-originated captive macaques (Captive, *n* = 43) captured from Khaoson-Samae Dam (KS), Bangkok (GPS:14° 34’N, 101° 08’E) permitted by the Department of the National Parks, Wildlife and Plant Conservation; permission no. 0909.302/5369 (25 Mar 2014) and 0909.702/1431 (25 Jan 2016).

The PPT colony was wild caught and specimens were collected onsite, while the KS were captured and transferred to NPRCT-CU for one year prior to the date of sample collection. These macaques were reared following standard animal biosafety guidelines. They lived in semi-opened gang cages and were fed twice a day; in the morning with standard macaque chow (Perfect Companion Group, Thailand) and in the afternoon with fresh fruits and vegetables. The age of macaques was estimated based on dental eruption pattern described previously [31]. All macaques were tested for herpes B virus infection specific antibodies using simian herpes virus ELISA test kit (VRL, Suzhou, China) in order to rear herpes B virus-positive and negative macaques separately. Male and female macaques of mature age, with or without herpes simian B virus were included in this study. All macaques were TB (Tuberculosis) negative and healthy with no apparent signs of illness. Additional characteristics and background information are described in Supplementary File 1.

### 2.2. Specimen Collection

The fecal swab samples were collected by veterinarians of NPRCT-CU. Samples from wild macaques were collected on the day of capture, while samples from captive macaques were taken during annual health check-ups. The macaques were anesthetized to reduce pain and distress during samples collection. The swabs were preserved in 15 mL conical tube containing 3 mL of viral transport media (VTM) and transported at 4 °C. The VTM was the mixture composed of 1× Hanks balanced salt solution (HBSS), 1% (*w/v*) of bovine serum albumin, 15 μg/mL of amphotericin B, 100 U/mL of penicillin G, and 50 μg/mL of streptomycin. The samples were processed within 12 h of collection. The experimental protocol of this study was approved by the Animal Care and Use Committee of the NPRCT-CU (Protocol Review No. 1775005; 28 Sep 2017)**.**

### 2.3. Viral Enrichment and Extraction

To reduce possible batch effects, samples were processed in an inter-digitated manner using one sample from a wild macaque followed by one from a captive animal etc. until all samples were processed for sequencing. Swabs were stirred in VTM and removed from the collection tube. The fecal suspension was centrifuged at 8000× *g* for 5 min at 4 °C and the supernatant (500 μL) was filtered through a 0.45 µm spin column filter (Millipore, Burlington, MA, USA) to remove bacteria and other large particulates. The flow-through was treated with a mixture of nuclease consisting of 400 µL of fecal filtrate, 14 U of Turbo DNase (Ambion, Thermo Fisher, Waltham, MA, USA), 3 U of Baseline-ZERO (Epicentre, Charlotte, USA), 30 U of Benzonase (Novagen, Darmstadt, Germany) and 30 U of RNase One (Promega, Madison, WI, USA) in 1× Turbo DNase buffer (Ambion, Thermo Fisher, Waltham, MA, USA). The reaction was incubated at 37 °C for 1.5 h and then extracted immediately using the MagMAX^TM^ Viral RNA Isolation kit (Applied Biosystems, Thermo Fisher, Waltham, MA, USA).

### 2.4. Reverse Transcription and Random Amplification of Viral Genome

The cDNA synthesis and random amplification were performed according to Li et al., 2015 [32]. Briefly, the 11 µL of extracted viral nucleic acid was annealed with 100 pmol of Sol A random primer: 5′-GTT TCC CAC TGG AGG ATA NNN NNN NNN-3′ (Eurofins Genomics, Louisville, KY, USA) at 72 °C for 2 min and 4 °C for 2 min. After that, the 20 µL reaction of first-stranded cDNA synthesis containing 800 U Superscript III reverse transcriptase (Invitrogen, Thermo Fisher, Waltham, MA, USA), 0.5 mM of each deoxynucleoside triphosphate (dNTPs), 5 mM dithiothreitol, and 1X Invitrogen RT buffer, and 2 mM RiboLock^TM^ RNase Inhibitor (Invitrogen, Thermo Fisher, Waltham, MA, USA) was prepared. The reaction was then incubated at 25 °C for 5 min, 50 °C for 60 min and 70 °C for 15 min, 4 °C for 5 min, 95 °C for 2 min and held at 4 °C. Next, the second strand cDNA synthesis was immediately performed by adding 5U Klenow Fragment DNA polymerase (New England Biolabs, Ipswich, MA, USA) into reaction and incubated at 37 °C for 60 min and 75 °C for 20 min. cDNA product can be stored in −20 °C until used. The cDNA was random-amplified by using Sol B primer: 5′-GTT TCC CAC TGG AGG ATA-3′ (complementary of 18 bp at 5′end of sol A primers). The reaction (25 µL) contained with 2 µM of Sol B primer, 1.85U of AmpliTaq Gold^®^ DNA Polymerase (Applied Biosystems, Thermo Fisher, Waltham, MA, USA), 0.25 of mM dNTPs, 4 mM of MgCl2, 1× PCR Buffer, and 5 µL of cDNA template. The thermal profile for amplification was composed of 95 °C for 5 min, 5 cycles of 95 °C for 30 s, 59 °C for 60 s, and 72 °C for 90 s, 35 cycles of 95 °C for 30 s, 59 °C for 30 s, and 72 °C for 90 s (+2 s per cycle) followed by 72 °C for 10 min and hold at 4 °C. The amplified product was checked by gel electrophoresis (approximately expected size 300–1000 bp).

### 2.5. Virome Library Preparation and Sequencing

The random amplified products were quantified by Quant-iT™ DNA HS Assay Kit using Qubit fluorometer (Invitrogen, Thermo Fisher, Waltham, MA, USA). by using Nextera XT DNA Library Prep Kit (Illumina, San Diego, CA, USA) with dual barcoding following the manufacturer protocol. Briefly, 3.5 µL of input DNA was mixed with 2.5 µL of Tagment DNA (TD) buffer and 1.25 µL of Amplicon Tagment Mix (ATM) and then incubated at 55 °C for 7 min and hold at 10 °C. The 1.25 µL of Neutralize Tagment (NT) Buffer was immediately added to stop tagmentation. The reaction was incubated at room temperature for 5 min and then added with 3.75 µL of Nextera PCR Mastermix (NPM) and 1.25 µL of each multiplexing index primers was added into reaction. The amplification was performed using the thermal condition of 72 °C for 3 min, 95 °C for 30 s, 15 cycles of 95 °C for 10 s, 55 °C for 30 s, and 72 °C for 30 s, and 72 °C for 5 min. The prepared library was checked by gel electrophoresis. The size of products ranged between 400–600 bp. After that, the concentration of DNA libraries was measured by Quant-iT™ DNA HS Assay Kit. The libraries were pooled at equal concentration and purified by Pippin Prep automated size selection machine based on 1.5% agarose gel electrophoresis. The purified library was quantified again by using KAPA library quantification kits for Illumina platforms (Kapa Biosystems, Wilmington, MA, USA). Then, 2 nM of library was denatured and loaded with the concentration of 10 pM with 20% PhiX into MiSeq sequencing platform for 2 × 250 cycles pair-end sequencing.

### 2.6. Data Processing and Analysis

Human reads and bacterial reads were identified and removed by comparing the raw reads with human reference genome hg38 and bacterial genomes release 66 (collected from ftp://ftp.ncbi.nlm.nih.gov/blast/db/FASTA/, Oct. 20, 2017) using Bowtie2 [33] in local search mode. The filtered sequences were deduplicated if base positions 5 to 55 were identical. One random copy of duplicates was kept. The sequences were then quality trimmed and trimmed for adaptor and primer sequences by using VecScreen [34]. After that, the reads were de novo assembled by EnsembleAssembler [35]. Assembled contigs and all singlet reads were aligned to in-house viral protein database (collected from ftp://ftp.ncbi.nih.gov/refseq/release/viral/, Oct. 20, 2017) using BLASTx (version 2.2.7) using E-value cutoff of 0.01 to identify highly divergent viral sequences. The significant hits to viruses were then aligned to an in-house non-virus-non-redundant (NVNR) universal proteome database using DIAMOND [36]. Matches with more significant E-values to NVNR than to the viral database were removed to eliminate false viral hits. The genome coverage of the target viruses was further analyzed by Geneious R7.0.6 software (Biomatters, Auckland, New Zealand).

### 2.7. Genome Acquisition of Novel Picornavirus

Two regions of ambiguous picornavirus sequences were re-sequenced by using two primer sets. The first region located on the 3′ end of the genome was amplified by 4F: 5′-GGA TAC TTT GTT TAG CTT TGC AAT TCT-3′ and 1,274R: 5′-ACA TGG TTC ATA GCC TGC CA-3′ for the first round and 65F: 5′-TTG TAG GGC GAC AGG TGT TC-3′ and 955R: 5′-AGC ACA ACT TGA TGA GGG CA-3′ for the second round of PCR. The second region in the middle of genome was amplified by 2,497F: 5′-TGC GTG GCG ATA TTT GCT TG-3′ and 4,553R: 5′-ACA CGA GAA GCA TGA GCC AT-3′ for the first round and 2,776F: 5′-CCT CAC AAG GAC GTG CTT GT-3’ and 4,005R: 5′-GAG CAG CAA CAG ACT TCC CA-3′ for second round of PCR. The amplification step composed of 95 °C for 5 min, 40 cycles of 95 °C for 30 s, 52 °C or 54 °C (for the first round or second round, respectively) for 30 s, and 72 °C for 90 min, and then 72 °C for 5 min. Amplicons were run on a 2% agarose gel and confirmed by Sanger sequencing. In order to sequence the missing 3′ region of novel picornavirus genome, the SMARTer^®^ RACE 5′/3′ Kit (Takara Bio USA, Inc., Mountain View, CA, USA) was used for 3′ Rapid Amplification of cDNA End (3′ RACE). Briefly, the cDNA synthesis was performed by using the 3′-CDs Primer A following the manufacture’s instruction. Then, cDNA was amplified by PCR using the primer Cyno_pico_3UTR_5,394F: 5′-GCC TGT CAT TCT GGG GAT ACAT TCT GCA GGA AAT GG-3′ and universal primer mix (UPM) provided in the RACE kit. The PCR thermal profile was 40 cycles of denaturation at 94 °C for 30 s, annealing at 67 °C for 30 s, and extension at 72 °C for 120 s, with a final extension step at 72 °C for 10 min. The PCR product was purified by QIAquick PCR Purification Kit (Qiagen, Hilden, Germany). Finally, the picornavirus sequencing data were processed and assembled using Geneious software. The cleavage sites of polyprotein were predicted based on the sequence alignment of picornaviruses in Supergroup 1 and cleavage sites reported in a previous study [37]. The structure of the internal ribosome entry site (IRES) was predicted by using the mfold web server [38] and illustrated by Forna web app [39]. The IRES type was classified based on predicted structure as described in previous studies [40,41].

### 2.8. Genome Completion of The Novel Parvovirus

A region with low coverage, located in the middle of the genome, was confirmed by nested PCR and Sanger sequencing. The primer for first PCR were Cyno_Parvo_1,273F: 5′-TGA AGG TGC TGG TAG CTT GG-3′ and Cyno_Parvo_2,827R: 5′-AAA CTG CCT CTC CTT CGT GG-3′ while the second PCR primers were Cyno_Parvo_1,438F: 5′-TGC CAT CCT GTG TTG ACT GA-3′ and Cyno_Parvo_ 2,590R: 5′-TCA GGG GAA TGT TCG ACA CG-3′. The amplification profile contained 95 °C for 5 min, 40 cycles of 95 °C for 30 s, 52 °C or 54 °C (for the first round or second round, respectively) for 30 s, and 72 °C for 90 min, and then 72 °C for 5 min. The open reading frame of the revised genome was then predicted by Open Reading Frame (ORF) Finder web application and compared with ORFs of known viruses. The splicing patterns of viral RNA were predicted by using neural network-based applications of the Berkeley Drosophila Genome Project (https://www.fruitfly.org /seq_tools/splice.html). Finally, the polyadenylation signals were predicted by SoftBerry POLYAH web application (http://www.softberry.com/berry.phtml).

### 2.9. Phylogenetic Analysis

The pairwise alignment of amino acid sequences and pairwise similarity calculation were performed by using CLUSTALW [42] implemented in Geneious software. The phylogenetic tree of P1, 2C and 3CD regions of picornavirus were constructed by MEGA X software [43] using the maximum likelihood method with Le_Gascule_2008 model 159 (LG) with gamma-distributed, and invariant sites (G + I). For parvovirus, the phylogenetic trees of the NP1 and VP1 regions were constructed by the maximum likelihood method using the LG model with invariant sites (I) and the general reverse transcriptase + freq. model, respectively. The phylogenetic relationship of circular Rep-encoding single-strand (CRESS) DNA virus was investigated by maximum likelihood method based on LG model. The statistical analysis of constructed trees was performed by calculation of bootstrap values (based on 1000 replicates) and presented on each node.

## 3. Results

### 3.1. The Study Population

The study population was comprised of a wild (*n* = 35) and captive (*n* = 43) populations. The summary of macaque characteristics is shown in Table 1. Due to the occurrence of natural herpes B virus infections, the viromes of herpes B positive and negative macaques could also be compared. In order to account for possible confounding factors due to age or sex, the characteristics between populations and between groups were statistically compared. The average age of wild macaques and those that were herpes B positive was significantly higher than that of captive macaques and those that were herpes B negative (*p* < 0.05), respectively.

### 3.2. Virome Composition

**Figure 1:**
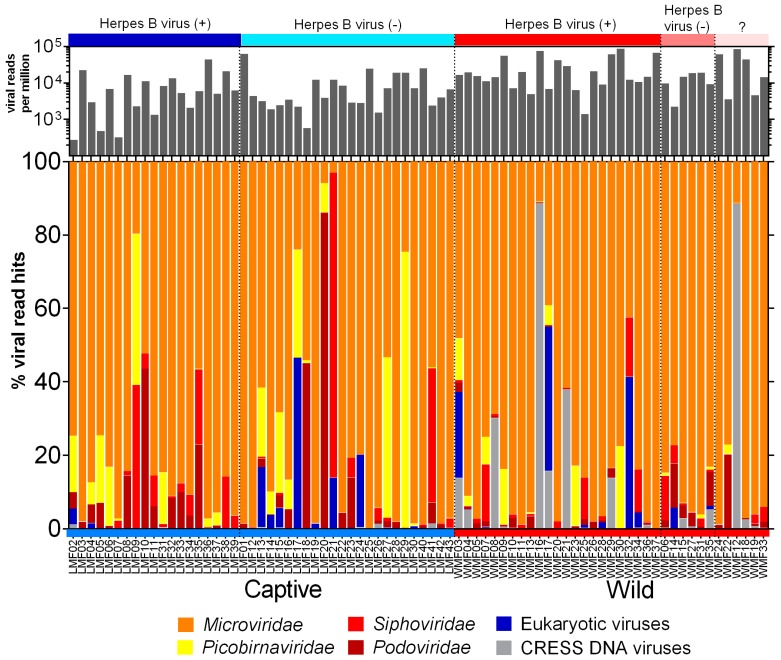
The percentage of viral reads matching six virus groups among wild and captive macaques. The color labels at the top of the figure present the herpes B virus infections. The black bar plot above the color bar shows the normalized total viral read numbers matching viruses shown below.

The raw sequence data generated from Illumina was processed using the in-house pipeline to deduplicate and filter low-quality sequences. The FASTQ sequences are available in the NCBI Sequence Read Archive (Bioproject: PRJNA548968). The summary of sequence processing is available in Supplementary File 2. The origins of the assembled contigs and unique reads from viral genomes were identified using BLASTx against an in house non-redundant viral protein database. Viral hits with an E value lower than 10^−10^ were used to determine relative abundance ((viral hits/total viral hits) ×100) and are shown in Figure 1. Alignments with proteins consisting of multiple short sequence repeats were considered artifacts and not counted. Reads from three families of phages (*Microviridae*, *Siphoviridae,* and *Podoviridae*) and one likely prokaryotic family (*Picobirnaviridae)* accounted for >90% of viral hits. The most common phage reads belonged to the *Microviridae* family, a rapidly expanding viral group [44], that contributed to over 70% of total viral reads. *Picobirnaviridae* reads were also abundant in a subset of captive macaques. While initially believed to be eukaryotic viruses recent studies indicated they are likely to be prokaryotic viruses [45,46]. Eukaryotic viruses (as defined below) were detected in both wild and captive cohorts.

### 3.3. Eukaryotic Viruses

**Figure 2:**
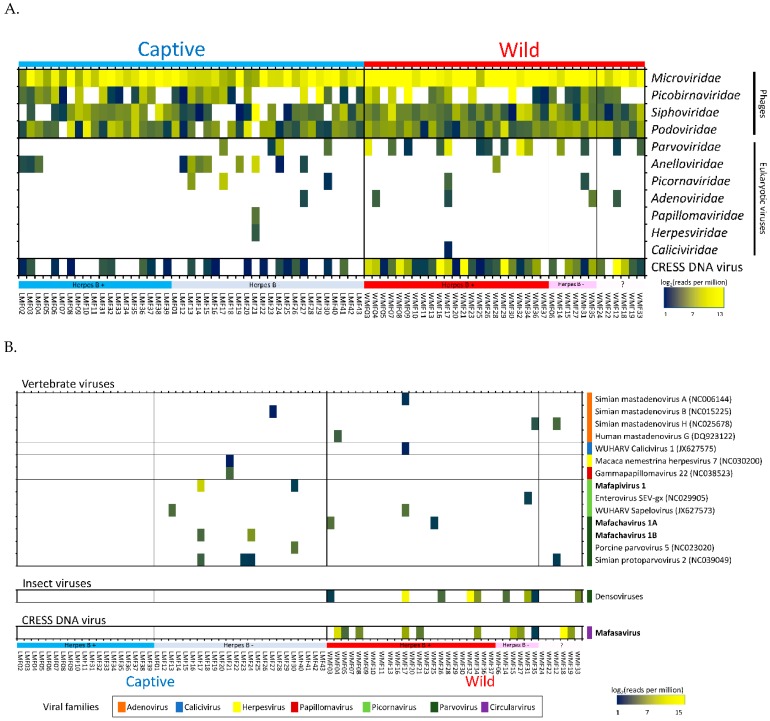
(**A**) Heatmap showing the relative abundance of viral reads identified by BLASTx into families. The families of viruses are grouped by host types; phage, eukaryotic viruses and circular Rep-encoding single-strand (CRESS) DNA virus. (**B**) Heatmap showing quantification of reads matching viral genomes from GenBank or contigs generated here (in bold). *Mafapivirus: Macaca fascicularis picornavirus* 1, *Mafachavirus: Macaca fascicularis chapparvovirus, Mafasavirus: Macaca fascicularis stool associated virus*.

Seven families of eukaryotic viruses were identified including members of the families *Parvoviridae*, *Anelloviridae*, *Picornaviridae*, *Adenoviridae*, *Papillomaviridae*, *Herpesviridae*, and *Caliciviridae* (Figure 2A). Members of the *Parvoviridae* and *Anelloviridae* families yielded the most reads. Anellovirus detection was more common in captive macaques (*n* = 11 captive, *n* = 1 wild) while parvovirus detection was more common in wild macaques (*n* = 14 wild, *n* = 4 captive). Most of the *Parvoviridae* reads in wild macaques belonged to the *Densovirinae* subfamily known only as insect-tropic viruses therefore representing likely dietary intake. Picornaviruses were found in three captive macaques (6.97%) and two wild macaques (5.71%). Adenoviruses were found in a single captive (2.33%) and four wild macaques (11.4%) with low read numbers. Other families detected (papillomavirus, herpesvirus, and calicivirus) were each found in a single sample. One animal in the captive group was shedding in its feces both papillomavirus and herpesvirus DNA. Lastly, a calicivirus was found in one wild macaque with very low read numbers.

The eukaryotic viruses found in these fecal viromes were therefore comprised of enteric viruses, respiratory viruses, invertebrate viruses, and viruses of unknown tropism. Not included in Figure 1 were reads matching plant-infecting viruses in the *Virgaviridae* family that were detected in only two macaques making up 91 and 3.6% of their identifiable viral reads.

Both previously described as well as novel viral genomes were detected. Reads matching different virus species were de novo assembled into larger, partial or full-length genome contigs. These contigs were then used to quantify the number of matching reads using the aligner program Bowtie2 (95% identity over at least 50 bases). When too few reads from a virus were generated to generate a contig we used the closest matching genomes from GenBank to quantify matching reads. The resulting proportion of matching reads are shown using a heat-map in Figure 2B. This mammalian virus distribution analysis showed that the group of captive herpes B virus-positive macaques did not yield any mammalian virus beside anelloviruses (Figure 2A,B). In contrast, other macaque groups shed several mammalian viruses together with anelloviruses.

Four species of mastadenoviruses were found in macaque samples. They included three strains of simian mastadenoviruses (A, B, and H) and of human mastadenovirus G. All of the reads aligned to the reference protein sequences with >95% amino acid identity. However, the number of hits were low (ranging from 1 to 69 reads per sample) possibly due to the generally respiratory tropism of this viral family. Adenovirus were each found in a single animal except simian mastadenovirus H found in two wild macaques. Another virus related to WUHARV calicivirus 1 was also detected (91.5% amino acid identity) with low read numbers in a wild macaque. This virus was originally found in feces of a captive rhesus macaque infected with SIV [8].

The herpesvirus contig (731 nt) found in this study was close to *Macaca nemestrina herpesvirus* 7 (91.2% amino acid identity) and detected in a single fecal sample of a captive macaque (LMF21). The same macaque also shed a papillomavirus showing a contig (660 nt) showing best match to *gammapapillomavirus 22* (82.5% amino acid identity). Both of these viruses have been associated with oral infections [47,48].

Numerous CRESS DNA virus reads were also detected with particularly high abundance in wild macaques. A complete CRESS-DNA genome was successfully assembled.

### 3.4. Detection of Three Picornaviruses

There are currently six supergroups of picornaviruses composed of 46 genera (www.picornaviridae.com). At least three picornaviruses were found in these Thai macaques (Figure 2B). A contig (435 nt) matching enterovirus SEV-gx (NC029905) was found in a wild macaque with 67.2% amino acid identity. This virus was first discovered in rhesus macaque in China. Another picornavirus matched the WUHARV sapelovirus (JX627573) originally reported in rhesus macaque with 96.7% amino acid identity. Contigs (max length = 733 nt) matching WUHARV sapelovirus were found in one captive and one wild macaque. The third picornavirus was a highly divergent novel picornavirus detected in two captive macaques further described below.

#### New *Picornaviridae* Genus

We characterized this novel picornavirus genome assembled from a captive macaque. BLASTx result showed this genome to be most closely related to viruses in supergroup 1, which includes the genera *Mosavirus, Aimelvirus, and Cardiovirus.* The coding sequences of the P1, 2C, and 3CD were compared to other members of supergroup 1. According to ICTV, the member of the same genus should share >33 % amino acid identity in P1 and >36 % amino acid identity in the non-structural proteins 2C + 3CD [49]. Here the novel picornavirus had P1 and 2C+3CD amino acid identity of 30.1% and 31.7% to the closest mosavirus and senecavirus relatives, respectively (Table S1) indicating that this virus may be classified into a new genus with a proposed genus name of *Mafapivirus* (*Macaca fascicularis picornavirus*; MfPiV) and proposed species name of *Mafapivirus 1* (MN312220).

**Figure 3:**
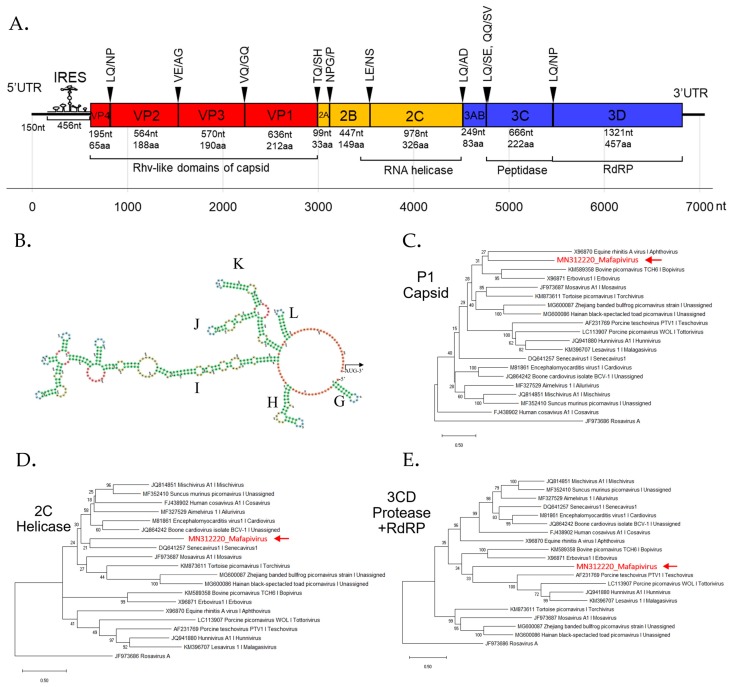
(**A**) Schematic genome organization of novel picornavirus (*Macaca fascicularis picornavirus 1* or *Mafapivirus 1*) identified from captive cynomolgus macaque (LMF17) indicating the cleavage sites and protein domains. (**B**) The predicted internal ribosome entry site (IRES) hybridization structure predicted by mfold software shows that this virus contains Type II IRES recognized by the conserved structure of Y-shaped J–K domains. Colors of the structure indicate the secondary structure in predicted IRES; Green: Stems (canonical helices), red: Multiloops (junctions), yellow: Interior loops, blue: hairpin loops, and orange: 5’ and 3’ unpaired region. The phylogenetic trees of *Mafapivirus 1* were plotted for (**C**) P1 region, (**D**) 2C region, and (**E**) 3CD region. The tree was drawn to scale, with branch lengths measured in the number of changes per amino acid site. Nodes in the tree represent % of bootstrapping value (1000 rounds).

The phylogenetic analysis of *Mafapivirus 1* based on P1, 2C, and 3CD amino acid sequences was performed with members of supergroup 1 as shown in Figure 3C–E. The analysis confirmed that this virus was highly divergent from all recognized genera as well as other, unclassified, picornaviruses.

The peptide domains in polyprotein were identified based on comparison to the NCBI Pfam database. The Rhv-like capsid domain, helicase, peptidase, and RNA-dependent RNA polymerase (RdRp) domains were found. Most polyprotein proteolytic cleavage sites were predicted based on similarity of previously reported picornavirus cleavage sites except for that between 3A and 3B proteins which could not be identified. The conserved NPG↓P motif that mediates co-translational termination-re-initiation of RNA translation was found between 2A and 2B peptides. The schematic diagram of the genome organization is shown in Figure 3A. This virus lacks leader (L) protein at 5′ region of polyprotein. The layout of peptide organization in polyprotein 2079 aa in length was 5′-(VP4-VP2-VP3-VP1/2A-2B-2C/3A-3B-3C-3D)-3′. The IRES structure of novel picornavirus belonged to Type II IRES. The signature conformation marked as I domain and Y-shaped J-K domain, the sites of eIF4G and eIF4A binding, are shown in Figure 3B.

### 3.5. Parvovirus

**Figure 4:**
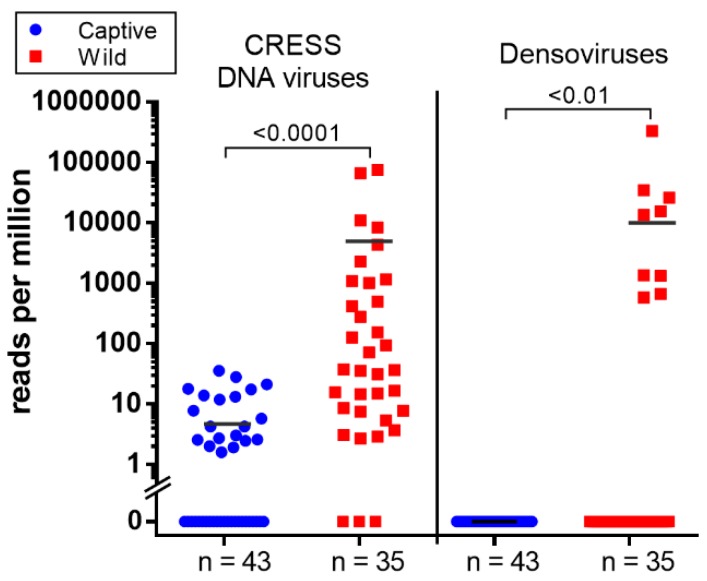
The scatter dot plot represents the relative abundance calculated as reads per million (RPM) of two group of viruses; CRESS DNA virus and densoviruses detected in wild and captive macaque. Both viruses show significantly higher abundance in wild group (Mann–Whitney *U* test; *p* < 0.05).

Parvoviruses are small, non-enveloped, linear, single-stranded DNA viruses found in a wide range of hosts. Their genomes are 4 to 6 kb in length and generally comprise two ORF encoding non-structural protein (NS) and viral structural protein (VP). Parvoviruses can be divided into two subfamilies; *Densovirinae* and *Parvovirinae*. Viruses in *Densovirinae* infecting invertebrate hosts are more diverse than *Parvovirinae*. We found densoviruses only in feces of wild macaques (see Figure 2B and Figure 4). Sequences matching densoviruses were related to *Diaphorina citri densovirus* and *Hymenopteran ambidensovirus* with >60% amino acid identity.

Four distinct parvoviruses member of the vertebrate-infecting *Parvovirinae* subfamily were detected. The complete coding sequences of one parvovirus was successfully assembled. We named it as mafachavirus 1A belonging to new species *Mafachavirus 1A* (*Macaca fascicularis chapparvovirus 1A*; MfChPV-1A) classified as a chapparvovirus. This virus was found in two wild macaques. In addition, we found another strain of this same virus with 91.3% nucleotide similarity in two captive macaques that was named mafachavirus 1B (MfChPV-1B). Different populations of Thai macaques therefore shed different chapparvovirus strains.

We also found 18 reads related to copiparvovirus and protoparvovirus in these macaques. The assembled contig (518 nt) of copiparvovirus matched porcine parvovirus 5 with 49.4% amino acid identity but we were unable to extend the contigs to full-length coding sequence. The protoparvovirus contigs (max length = 846 nt) in this study were closely related to simian protoparvovirus 2 (92.0% amino acid identity) and were detected in both wild and captive macaques.

#### The First Simian Chapparvovirus Genome with Complete ORFs

**Figure 5:**
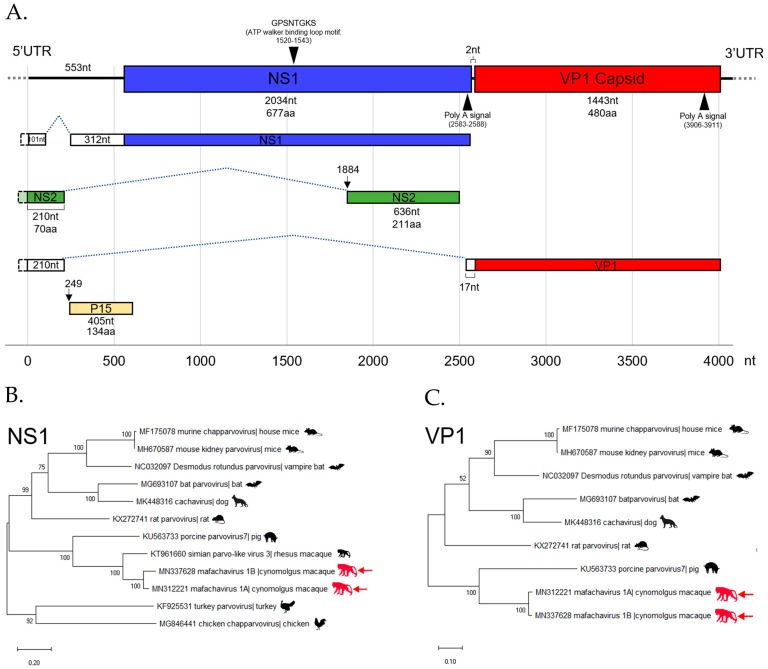
(**A**) The diagram shows the genomic structure and predicted splicing pattern of the novel chapparvovirus (*Macaca fascicularis chapparvovirus 1*; *mafachavirus* 1A) identified from wild cynomolgus macaque (WMF03). The assembled genome contains two major open reading frames (ORFs); non-structural protein 1 (NS1) and viral capsid protein 1 (VP1) and the sequence of NS2. The functional motif and Poly A signal are also illustrated. The phylogenetic trees of Mafachavirus 1A and 1B were constructed based on (**B**) the NS1 region and (**C**) the VP1 region. The trees include the reference sequences from other chapparvovirus members. Nodes indicate % of bootstrapping value (1000 rounds). The tree was scaled by the number of changes per amino acid site.

The mafachavirus 1A genome sequence was assembled from reads matching parvoviruses in a wild macaque (WMF03). A low-coverage region of partial genome was confirmed by nested PCR and Sanger sequencing. The 3′ end of genome was sequenced using 3′RACE. The sequence of the two major ORFs; NS1 and VP1 were generated. The genome of novel chapparvovirus is shown (Figure 5A). The NS1 gene encodes a 677 amino acids protein and the VP1 gene—a 480 amino acids long capsid protein. Two major ORFs were separated by a short 2 nucleotides region as typical of chapparvoviruses. The conserved P15 accessory protein (134 amino acids) was also recognized. Polyadenylation signals were identified near the 3′ end of expected NS1 and VP1 transcripts. In addition, the conserved ATP walker binding loop motif was found in NS1. Based on a recent study of chapparvovirus [50], we identified three alternative splicings of viral RNA. Both NS1 and VP1 transcripts are expected to be spliced onto a 5′UTR region RNA. In addition, we also found an additional small predicted NS2 transcript located within NS1 ORF but lacking a start codon indicating that splicing is required for translation. A putative upstream NS2 exon near 5′ end of genome matched with the recently reported NS2 sequences of mouse kidney parvovirus (YP009553676) [51] and simian parvo-like virus 1 (APC23171). These are key characteristics indicating that mafachaviruses 1A and 1B are members of type I exogenous amniote chapparvoviruses [50,51].

The mafachavirus NS1 protein is most closely related to partial NS1 sequence of simian parvo-like virus 2 with 91% amino acid identity (Figure S4.). However, simian parvo-like virus 2 partial genome submitted to GenBank (KT961661) lacked VP1 sequences [52]. Therefore, this is the first reported simian chapparvovirus genome with complete ORFs. The VP1 of mafachavirus sequenced here was distantly related to that of porcine parvovirus 7 with 54% amino acid identity. The ICTV guideline suggested that parvoviruses within a species should have >85% amino acid NS1 identity while different species within the same genus should have >30% identity amino acid identity of NS1. Following these guidelines, we purpose the species name of this virus as *Mafachavirus* 1 (MN312221) since pairwise NS1 similarities showed maximum amino acid identity of 46.6% when compared with porcine parvovirus 7 (Table S2.). The phylogenetic relationship of this novel chapparvovirus was also investigated using VP1 and NS1 proteins. The VP1 phylogenetic trees showed that porcine parvovirus 7 is the closest relative of mafachavirus 1A and 1B (Figure 5B). For the NS1 tree, the novel virus clustered with that of the complete NS1 of simian parvo-like virus 3 from *Macaca mulatta* in the US [52]. This indicated that the chapparvovirus we named mafachavirus in long-tailed macaque is closely related to chapparvovirus partial genomes originally reported from US captive rhesus macaques (simian parvo-like virus 1/KT961662, simian parvo-like virus 2/KT961661, parvo-like virus 3/KT961660) [52].

### 3.6. CRESS-DNA Virus

Circular Rep-encoding single-strand (CRESS) DNA virus make up a large and diverse group of small DNA viruses. Their general genomic structure consists of two genes encoding the replication-associated protein (Rep) and the nucleocapsid (Cap). The genome size varies among groups generally ranging from 1.7 kb to 3 kb. Here CRESS DNA virus-related proteins were more abundant and shedding prevalent in wild versus the captive macaque (Figure 2A and Figure 4).

#### Novel Stool-Associated CRESS DNA Virus

**Figure 6:**
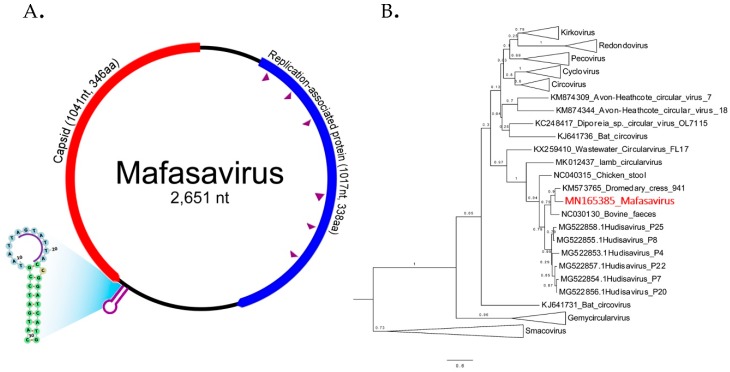
(**A**) The schematic diagram shows the genome organization of *Macaca fascicularis stool-associated virus* (species *Mafasavirus*) detected from wild macaque (WMF18). Two main ORFs; capsid (Cap) and replication-associated protein (Rep) were recognized. The purple arrowheads indicate the conserved motifs of Rep gene. The motif sequences are shown in supplementary data. The stem-loop origin of replication contains a canonical nonanucleotide sequence (AGTATTAC). (**B**) Phylogenetic Rep tree. The reference sequences in the tree include representative of different CRESS virus families and unclassified genomes. The tree was drawn to scale, with branch lengths measured in the number of changes per amino acid sites. Nodes indicate % of bootstrapping value (1000 replicates).

We characterized a complete genome of a CRESS DNA virus from wild macaque WMF03 (Figure 6A). The genome size was 2651 nucleotides containing the two main Rep and Cap ORFs arranged in opposite direction. The expected Cap protein was 346 amino acids while the Rep protein was 338 amino acids. The expected rolling circle replication motifs I, II, and III and superfamily 3 helicase Walker A, B, and C motifs within Rep protein were identified (sequences of motifs shown in Table S3.). The closest Rep relatives were Dromedary stool-associated CRESS virus (62.8% aa identity) and bovine feces-associated CRESS virus (55.6% aa identity).

The phylogenetic relationship of this genome was investigated by comparison of Rep protein among representatives of CRESS virus genera shown in Figure 6B. Rep sequence clustered with two unclassified CRESS viruses named Dromedary stool-associated CRESS virus (62.8 % amino acid identity) and Bovine feces-associated CRESS virus (55.6 % amino acid identity). The next closest Rep sequences were from hudisaviruses sequenced from feces of humans and other mammals [53]. We named the novel CRESS virus species “*Mafasavirus*” (*Macaca fascicularis stool-associated virus*; MfSaV; MN165385).

## 4. Discussion

Phages can contribute to the modulation of bacterial populations in the gut and regulation of intestinal physiology [2,54]. The characterization of fecal swab viromes from 78 cynomolgus macaques showed that bacterial virus reads contributed the largest fraction of recognizable viral reads. Fecal virome composition may also be influenced by diet [55,56,57]. Changes in virome could affect the gut bacterial populations and contribute to digestive tract diseases such as inflammatory bowel disease [21,58]. Here the most prevalent phage reads belonged to members of the *Microviridae* family, believed to infect mostly Bacteroidetes [59]. The *Microviridae* has experienced a large increase in its recognized diversity and specific member do not appear to be associated with particular mammals [44,60,61].

We focused here on the eukaryotic virome and compared wild and captive animals with or without concurrent herpes B specific antibodies. It was found that the herpes B virus sero-positivity was more prevalent in older macaques since it is typically transmitted through copulation or fighting [62]. Overall seven families of eukaryotic viruses were identified. We detected anelloviruses at the highest frequency in the captive monkeys testing sero-negative for herpes B virus (34.8%) which is also the youngest group of animals. Anellovirus was detected in only three herpes B sero-positive captive (15.8%) and a single herpes B positive wild animal (2.85%). Increased anellovirus viral loads have been associated with reduced levels of host immunocompetence. [63,64,65,66] Other mammalian viruses from five families (*Parvoviridae*, *Picornaviridae*, *Adenoviridae*, *Papillomaviridae*, and *Herpesviridae*) detected in captive herpes B sero-negative were not detected in older captive herpes B sero-positive animals. The significantly younger age of the herpes negative versus positive captive animals may also account for their higher rates of enteric viral infections [67].

Shedding of herpes B usually occurs when macaques are immunosuppressed or stressed during mating season [68]. Another herpesvirus (*Macaca nemestrina herpesvirus 7* or MneHV7) was shed by one animal. MneHV7 is roselovirus in the group of betaherpesvirus infecting salivary gland of macaques [47] and is the viral homolog of human herpesvirus 7 (HHV7) which can be detected in the human oral cavity [47]. The same animal was also infected by a gammapapillomavirus related to oral tissue-infecting gammapapillomavirus 22 or human papillomavirus 172 [48] suggesting MneHV7 and gammapapillomavirus co-infection, possibly in its oral cavity, followed by fecal shedding.

As previously reported for other non-human primate species [69,70] several adenoviruses were also detected including adenovirus G in one macaque. The species-crossing capacity of adenoviruses has been demonstrated [70,71,72,73,74]. Adenovirus G has also been reported in *Macaca assamensis* from Thailand [70]. Whether the adenovirus G virus detected here has the potential to infect human remains to be determined but represent the virus closest in sequence to one known to infect humans.

We identified several eukaryotic viral sequences closely related to those of previously reported rhesus macaque viruses. For example, we found viruses related to WUHARV calicivirus, WUHARV sapelovirus, and WUHARV parvovirus originally discovered in rhesus macaques infected with simian immunodeficiency virus housed in New England Primate Research Center (NEPRC) [8]. Another virus identified in a wild cynomolgus macaque is a close relative of enterovirus SEV-gx reported from rhesus macaque in China (NC029905). Even though several macaque viruses were identified there was no support for their pathogenicity as all were collected from apparently healthy animals.

We also characterized the first genome including all ORFs of a simian chapparvovirus including NS1, NS2, VP1, and p15 (in *Mafachavirus 1* species) This genome is a close relative of previously reported parvoviruses named simian parvo-like viruses which did not include VP1 sequence from the feces of rhesus macaques from the California National Primate Research Center [52]. These closely related chapparvoviruses (91% identity in partial NS1 and therefore likely in same viral species) were therefore shed by two different macaque species (*M. mulatta* and *M. fascicularis*). Different strains of mafachaviruses were also identified in wild and captive Thai macaques suggesting circulation throughput the Thai macaque population. *Mafachavirus* is the sister taxa to porcine parvovirus 7 detected in swine populations from several countries including China and Korea [75,76,77,78]. Reads from another parvovirus were also detected that were distantly related to porcine parvovirus 5 and 6 in the copiparvovirus genus reported in pig lung [76,79] that may represent a novel macaque copiparvoviruses. A full genome will be required to confirm this possibility.

The novel picornavirus characterized from a captive cynomolgus macaque was highly divergent and a member of a proposed new genus provisionally named *Mafapivirus*. This virus was detected in only two wild herpes B negative monkeys. Because no specific disease signs were reported in these two macaques this virus there is currently no evidence for its pathogenicity at least in immuno-competent animals.

We also found a different distribution of likely dietary viruses between wild and captive macaques particularly CRESS DNA viruses and densoviruses. The CRESS viruses detected in feces are highly divergent from those that have been recently classified. CRESS DNA virus has been identified from feces of human and numerous mammals including rhesus macaque and birds and in most environmental virome studies. [52,53,80,81] The only CRESS-DNA viruses which have been shown to replicate in mammals are members of the *Circoviridae* family infecting pigs, dogs, and other mammals [82,83,84]. The non-*Circoviridae* CRESS-DNA genomes found here are therefore of unknown tropism. The common detection of such genomes in fecal samples indicates that these viruses may be ingested rather than replicate in mammalian cells or may even replicate in gut parasites such as protozoa and nematodes [85]. The higher levels of CRESS-DNA read in wild versus captive macaques may reflect difference in their diet or prevalence of co-infecting parasites. The more common detection of densovirus DNA in feces of wild macaques may also reflect a greater consumption of insects by wild versus captive macaques. It is known that when fruits are unavailable for the generally frugivorous cynomolgus monkeys their diet will expand to include insects, leaves, seeds, mushrooms, invertebrates, and bird eggs [86]. Because samples were collected in the fruitless dry season, the densoviruses in wild macaque feces may be infecting consumed insects or insect contaminating other foods. The levels of densovirus DNA in feces may therefore reflect macaque habitats and diet. 

The characterization of fecal viromes in Thai cynomolgus macaques therefore identified several vertebrate viruses putatively infecting macaques including parvoviruses, anelloviruses, picornaviruses, adenoviruses, papillomaviruses, herpesviruses, and a calicivirus. Three genomes of novel viruses were characterized. Despite the viromes of wild cynomolgus macaques showing higher abundance of CRESS DNA viruses and densoviruses than those of captive animals the distribution of clearly mammalian viruses did not differ between these populations. Retention of a diverse mixture of enteric viruses after short term captivity therefore indicate that wild cynomolgus macaques reared in captivity for one year show no reduction in the complexity of their mammalian fecal virome. Virome analyses of feces from rhesus macaques (*Macaca mulatta*) born in captivity in US primate centers [52,87,88] also show the presence of numerous enteric viruses mimicking the situation in their wild counterparts [89].

Bites from wild and captive macaques are known to transmit both rabies and herpes B viruses to humans [90,91]. Rapid treatment with rabies vaccine and anti-herpes drug such as acyclovir are therefore recommended [92,93,94]. Whether any of the enteric viruses described here pose any risks to humans is currently unknown but detection of these viruses in symptomatic humans in recent contact with cynomolgus macaques will be required to substantiate such theoretical risk.

## Figures and Tables

**Table 1:** Basic characteristics of macaque samples in viral metagenomic sequencing.

Characteristics			All	Herpes B Virus-Positive (+)	Herpes B Virus-Negative (−)	*p*-Values (Herpes B Positive vs Negative)	*p*-Values (Captive vs Wild)
Age (years) ^a^	captive		3.34 ± 1.65	4.68 ± 1.40	2.27 ± 0.88	<0.001 ***	<0.05 *
	wild ^c^		5.48 ± 4.11	6.14 ± 4.27	2.75 ± 1.31	<0.01 **	
Sex (%) ^b^	captive	M	20 (46.5%)	9 (20.9%)	11 (25.6%)	0.74	0.24
		F	23 (53.5%)	10 (23.3%)	13 (30.2%)		
	wild ^c^	M	19 (61.29%)	16 (51.61%)	3 (9.68%)	0.53	
		F	12 (38.71%)	9 (29.03%)	3 (9.68%)		
Bodyweight ^a^	captive		3.13 ± 1.21	3.95 ± 1.04	2.49 ± 0.91	<0.001 ***	<0.05 *
	wild ^c^		4.16 ± 1.90	4.05 ± 1.90	2.78 ± 1.11	<0.05 *	
Crown–Rump Length ^a^	captive		386.6 ± 58.4	428.4 ± 45.8	353.5 ± 44.4	<0.001 ***	<0.05 *
	wild ^c^		415.4 ± 55.5	425.5 ± 51.9	371.5 ± 47.8	0.053	

^a^ (Mean ± SD), statistical significance tested by *t*-test: * *p* < 0.05, ** *p* < 0.01, *** *p* < 0.001; ^b^ number (%), statistical significance tested by the Fisher’s exact test; ^c^ Six wild macaques were not included in table due to a lack of herpes B test results.

## Supplementary Materials

The following are available online at https://www.mdpi.com/1999-4915/11/10/971/s1, Table S1: The percentage of amino acid identity between novel picornaviruses and closely related picornaviruses in P1, 2C + 3CD regions, Table S2: The pairwise amino acid identity of NS1 protein between novel mafachavirus 1A and closely related chapparvovirus, Table S3: The sequence conserved functional motifs within Rep protein of *Macaca fascicularis stool-associated virus* (*Mafasavirus*), Figure S4: The alignments of viral contigs from *Macaca fascicularis* compared to the reference sequences of viruses from NCBI, Supplementary File 1: The metadata of macaque samples, Supplementary File 2: The summary table of reads processing and data analysis.
